# The NtAN3 gene plays a key role in increasing tobacco leaf area

**DOI:** 10.1016/j.bbrep.2026.102749

**Published:** 2026-08-13

**Authors:** Shuxian Yang, Lili Duan, Renxiang Liu

**Affiliations:** a College of Tobacco, Guizhou University, Guiyang, 550025, PR China; b Guizhou Provincial Key Laboratory for Tobacco Quality Improvement and Efficiency Enhancement, Guiyang, 550025, PR China; c Journal of Guizhou University, Guiyang, 550025, PR China

**Keywords:** Tobacco, Leaf area, Transcriptomics

## Abstract

Tobacco (*Nicotiana tabacum* L.) is an important cash crop, and the size of its leaves significantly influences both yield and quality. However, previous research often focused on cultivation measures to enhance yield and quality, while the study of the structure and function of genes at the molecular level has been overlooked. We measured the leaf areas of *NtAN3* overexpressing mutants, silenced mutants, and wild type. Compared with the wild type, the leaf area of the overexpressing mutant was significantly increased, and the leaf area of the silenced mutant was significantly decreased, indicating that *NtAN3* plays a role in regulating the leaf area of tobacco. Cytological observations based on paraffin sections revealed that *NtAN3* positively modulates leaf cell proliferation and expansion at the early developmental stage, thereby determining the final leaf area of tobacco. To further explore the downstream transcriptional changes triggered by altered *NtAN3* expression, we performed comparative transcriptome sequencing using leaf samples collected at the flower budding stage. We selected two mutants, G27 and M21, which have significant differences in leaf area, and their parents for transcriptome analysis. There were 1697 differentially expressed genes in WT vs G27 and 2388 differentially expressed genes in WT vs M21. Functional enrichment showed remarkable transcriptional variations in carbohydrate metabolism pathways. Further analysis of starch, sucrose and galactose metabolism demonstrated that upregulated genes in these pathways mediate secondary metabolic adjustments. In addition, Nitab4.5_0000564g0140, Nitab4.5_0006318g0030, and Nitab4.5_0002093g0100 participate in late leaf metabolic responses induced by *NtAN3*. This transcriptomic research enriches our knowledge of the complete tobacco leaf regulatory network and provides molecular references for high-yield tobacco breeding.

## Highlights

• We generated tobacco *AN3* mutants , transcriptomics dissected genetics of leaf phenotypic variation.
• It uncovered the role of carbohydrate synthesis in tobacco leaf phenotypic regulation.
• *AN3* expression influences the expression of genes related to cell size.

## 1 Introduction

Tobacco (*Nicotiana tabacum* L.) is an economic crop harvested for its leaves. The size and shape of the leaves jointly determine the plant's effective photosynthetic area, which influences the yield and quality of photosynthetic products per leaf [1]. However, the dense tissue structure and narrow, thick leaves of upper tobacco leaves severely impair their yield and quality, resulting in low usability of upper tobacco leaves [2,3]. *AN3* (ANGUSTIFOLIA3) is an important transcription co-activator in plants and plays a crucial role in leaf area development [4]. Research has shown that *AN3* plays a key role in regulating cell proliferation and leaf primordia expansion. In Arabidopsis, overexpression of *AN3* increases leaf width, leading to larger leaf area, while *AN3* functional deficiency or mutation results in reduced cell numbers, causing narrower leaves and smaller leaf area [[4], [5], [6], [7]]. In recent years, transcriptomic studies have provided a molecular basis for leaf area development in Arabidopsis [4] and Chinese cabbage [8]. However, differences still exist in the expression patterns of *AN3* gene and the interaction mechanisms of its upstream and downstream regulatory networks across different crops.

The growth and development of plant leaves is a complex process involving a large number of genes. Research shows that leaf morphology is influenced by a variety of genetic factors, such as plant hormones, transcription factors, and miRNAs [5,7,9]. In addition, there is a close relationship between leaf development and carbohydrate metabolism [10].Sucrose and starch metabolism are important components of carbohydrate metabolism [11]. Sucrose is ultimately broken down into hexoses or their derivatives, which are used in various metabolic and biosynthetic processes. Research has shown that sucrose synthase (SUS) and invertase (INV) in plants can catalyze the breakdown of sucrose [12,13]. INV not only participates in primary carbon metabolism but also plays a regulatory role in plant growth and development [14].

Cell size plays an important role in leaf development [15]. The target of rapamycin (TOR) pathway plays a crucial role in plant growth and development [16]. In addition, it has been reported that *ABCB* plays an important role in auxin transport [17].Auxin-binding protein 1 (ABP1) is an auxin receptor that is closely related to plant development [18].

In tobacco, leaf area is a key indicator of plant growth and development, yield formation, and quality regulation. Previous studies have shown that cultivation practices such as planting density, number of leaves retained, and fertilization can improve leaf area size [[19], [20], [21]]. Since the AN3 gene in Arabidopsis can regulate leaf area at the molecular level [22]. Therefore, this study preliminarily revealed the role of the *AN3* gene in increasing tobacco leaf area and performed transcriptomic sequencing on mutant plants, followed by enrichment analysis of differentially expressed genes, providing new insights into the biological processes involving the synergistic effects of multiple genes in enlarging leaf area. These findings contribute to further elucidating the mechanisms underlying tobacco leaf development.

## 2 Results

### 2.1 Cloning and functional prediction of the tobacco *NtAN3* gene

Specific primers were designed based on the CDS sequence of the tobacco *NtAN3* gene, and *NtAN3* was subsequently amplified using PCR. Based on the physicochemical analysis of AtAN3 from *Arabidopsis thaliana* and tobacco NtAN3 proteins (Table 1), both proteins consist of 210 amino acids. NtAN3 has a molecular weight of 22491.79 Da and AtAN3 is 22463.62 Da. Their isoelectric points are 5.78 and 6.02 (both < 7), confirming that they are acidic proteins. The aliphatic indices are 54.90 and 55.38, reflecting a moderate level of aliphatic amino acids. The hydropathy indices are −0.80 and −0.72, and the instability indices are 71.50 and 67.13 for AtAN3 and NtAN3 correspondingly. These similar physicochemical properties suggest functional similarity between *AtAN3* and *NtAN3.*

**Table 1:** Tobacco *NtAN3* physical and chemical properties analysis.

proteins	Number of amino acids	Molecular weight	Isoelectrc point	Aliphatic index	hydropathy index	Instability index
AtAN3	210.00	22463.62	5.78	54.90	−0.80	71.50
NtAN3	210.00	22491.79	6.02	55.38	−0.72	67.13

Analysis of functional domains (Supplementary Fig. 3) revealed that both proteins harbor the SSXT domain and share structural similarities. Purple bars denote conserved amino acid regions, whereas grey lines represent non-conserved polypeptide sequences. In addition, predicting and analyzing the secondary structures of AtAN3 and NtAN3 proteins(Table 2) showed that AtAN3 and NtAN3 the proportions of α-helix were 39.52% and 36.67%; β-turn accounted for 8.10% in both proteins; extended strand occupied 13.33% and 14.76%, while random coil made up 39.05% and 40.48%. These data demonstrate a high degree of similarity in the secondary structures between AtAN3 and NtAN3. Meanwhile, we used thethree-dimensional structural models of AtAN3 and NtAN3 proteins were predicted using the Phyre2 online server (http://www.sbg.bio.ic.ac.uk/phyre2/html/page.cgi?id=index)(Supplementary Fig. 4), their tertiary structures also exhibited strong resemblance, further supporting the high functional similarity of these two proteins.

**Table 2:** Protein secondary structure analysis.

Proteins	Alpha helix	Extended strand	Beta turn	Random coil
AtAN3	39.52	13.33	8.10	39.05
NtAN3	36.67	14.76	8.10	40.48

### 2.2 Effect of *NtAN3* on tobacco leaf area

**Fig. 1:**
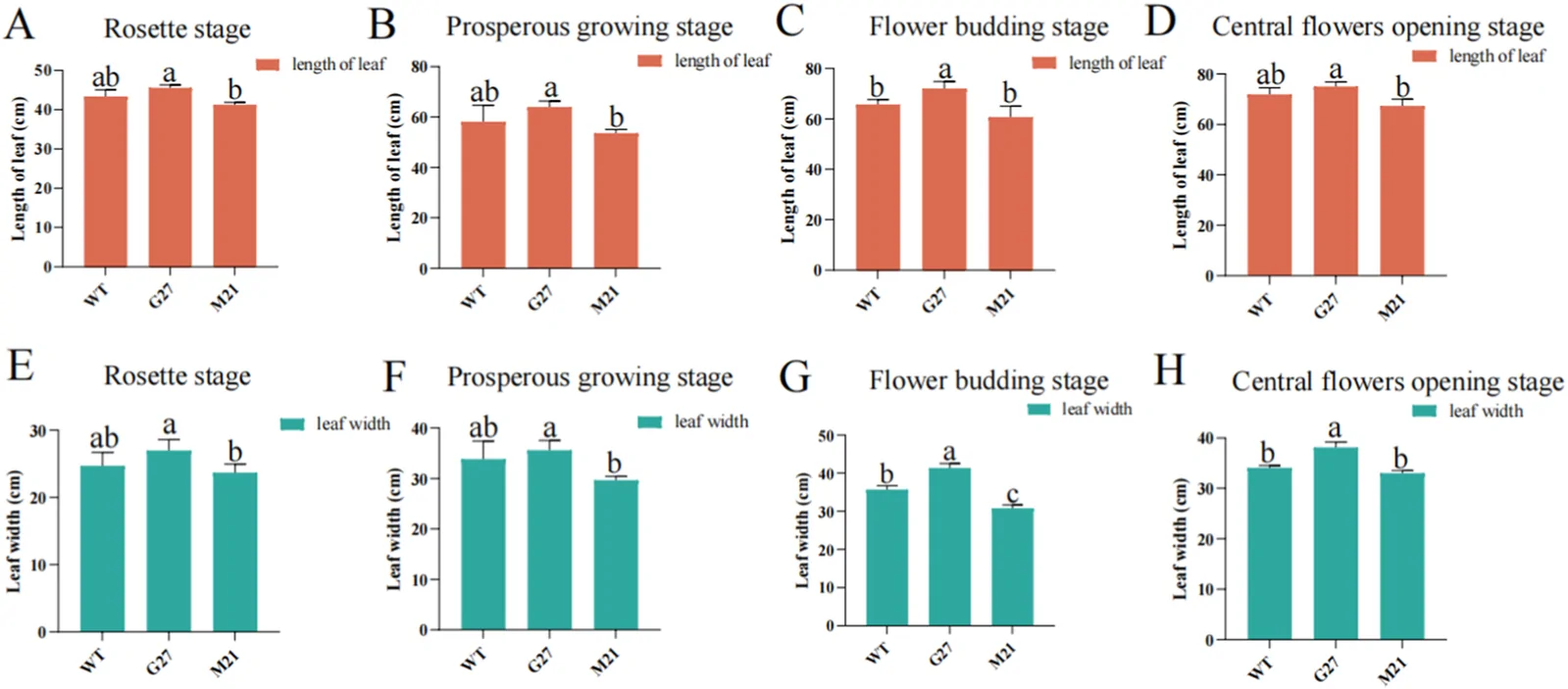
Leaf length and leaf width of the mutant and wild type at different developmental times. Note: All data are means of three biological replicates. Error bars indicate standard error. Different lowercase letters indicate significant differences at P < 0.05. Wild type: WT; overexpression mutant plants: G27; silencing mutant plants: M21.

To investigate the effect of the tobacco NtAN3 gene on tobacco leaves, we constructed NtAN3 gene overexpression mutant plants (G27) and silencing mutant plants (M21) and measured the leaf length and width of wild-type and mutant plants. Based on the developmental results of tobacco mutant leaf length and width at different stages (Fig. 1), The leaf length of mutant plants G27 and M21 showed significant differences during the rosette stage, prosperous growing stage, flower budding stage, and central flower opening stage. But for leaf length, the significant difference was only detected at flower budding stage, between WT and G27. In contrast, there were significant differences in leaf width between G27 and WT, and M21 and WT during the flower budding stage. This indicates that *NtAN3* promotes the growth of tobacco leaf length and width more during the flower budding stage. We then selected mutant plants in the flower budding stage for further analysis.

### 2.3 Sequencing analysis of mutant lines

Specific primers were designed to amplify the partial coding sequence (CDS) of endogenous tobacco NtAN3 for Sanger sequencing. PCR amplification was performed on positive mutant lines using primers designed flanking the target gene. The amplified products were digested with restriction enzymes, and the digested fragments were sent to a commercial sequencing company for sequence verification. Sequencing results are presented in Supplementary Fig. 1.

### 2.4 Analysis of target gene expression in mutant lines

Gene expression level in leaves serves as a direct indicator to quantify the abundance of the target gene in plant leaf tissues. Primers were designed based on the sequence of the target gene for quantitative real-time PCR (qRT-PCR) analysis of gene expression. Representative G27 and M21 were selected for expression quantification (Supplementary Fig. 2). Total RNA was extracted from tobacco leaf tissues using RNAiso Plus (Trizol) reagent following the protocols of the RNAprep Pure Plant Kit (TIANGEN, China). First-strand cDNA was synthesized with the FastKing gDNA Dispelling RT SuperMix kit (TIANGEN, China) according to the manufacturer's instructions. All procedures were performed on ice to avoid RNA degradation. The results revealed significantly altered transcription levels of the target gene in both mutant genotypes. Specifically, the transcript abundance was markedly higher in the overexpression mutant and considerably lower in the silenced mutant relative to wild-type plants. These findings confirm that the genetic modification effectively regulated the transcription of the target gene.

### 2.5 Cytological analysis

**Fig. 2:**
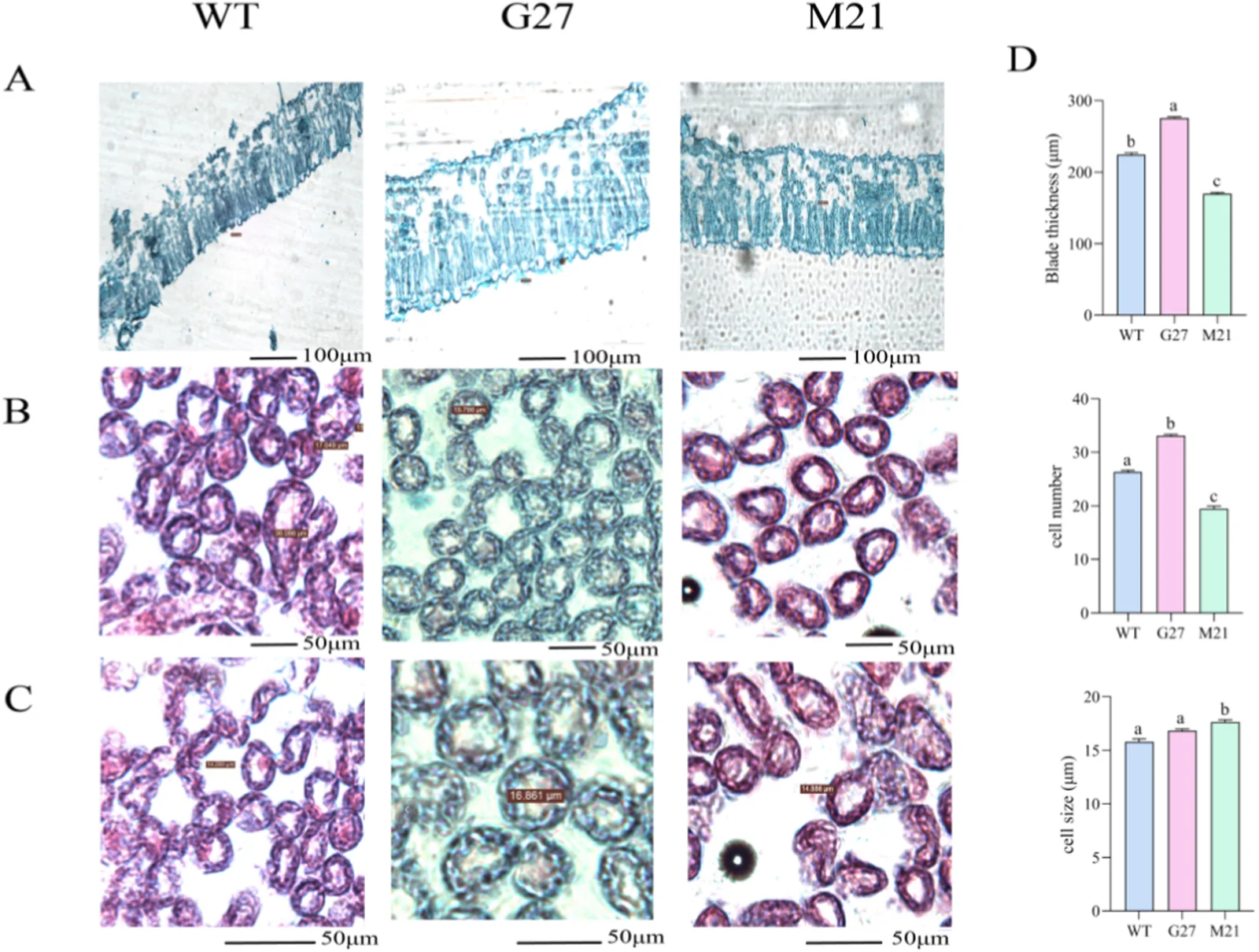
A:Blade thickness; B:Cell number; C:Cell size.D:Statistical chart. Note:Fig. 2C is a fixed-size statistical region cropped from panel Fig. 2B with equal magnification.

To further explore the cytological basis of leaf morphological changes, paraffin sectioning and microscopic observation of leaf tissues were performed(Fig. 2, Supplementary Fig. 5). Quantitative analyses of blade thickness, cell number and cell size were conducted based on leaf paraffin cross-sections across WT, G27 and M21 tobacco leaf. All original measurement data and statistical outcomes are compiled in Supplementary Table 4. Cytological analysis showed significant differences in cell structure between wild-type and mutant plants. The results showed *NtAN3* simultaneously regulates cell proliferation and cell expansion, and the elevation in cell number serves as the major contributor to leaf enlargement in overexpression plants.

### 2.6 Transcriptome analysis of leaf area in tobacco

**Fig. 3:**
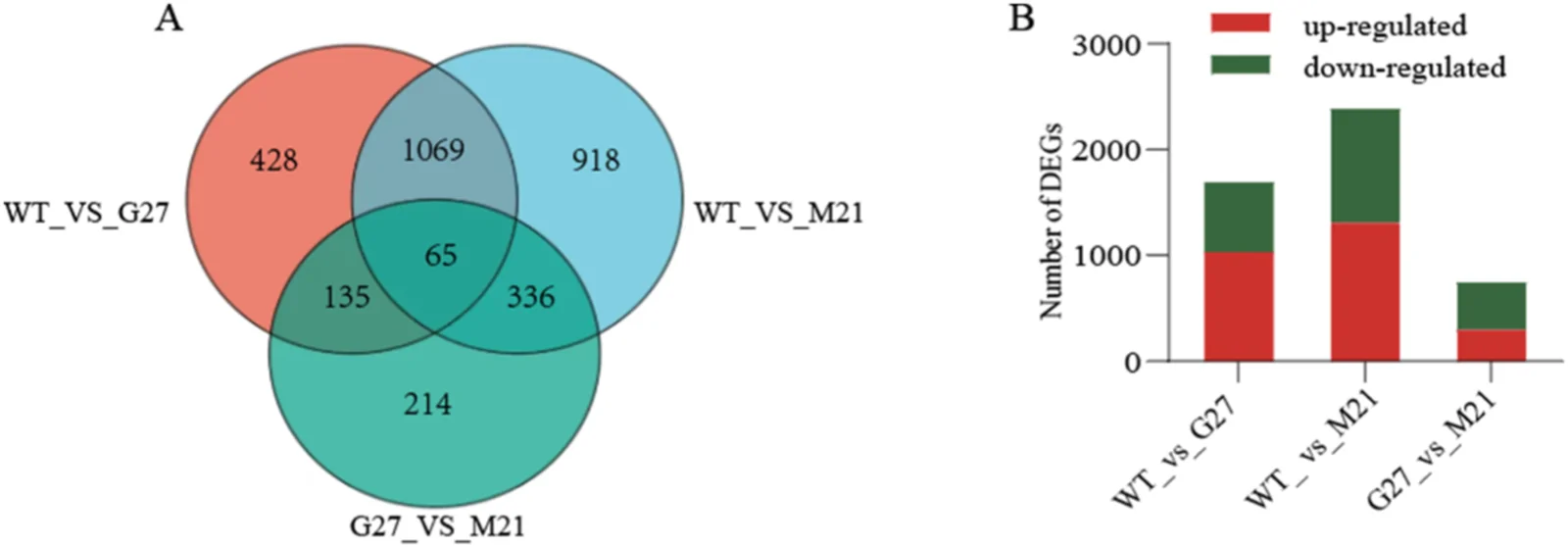
Distribution of differentially expressed genes (DEGs) between mutant plants and wild-type plants. (A) Venn diagram of differentially expressed genes (DEGs). (B) DEGs expression changes.

Using mutant plants G27 and M21 in the flower budding stage and their wild types as sequencing materials, 9 cDNA libraries were constructed and sequenced on the Illumina HiSeq Xten platform. A total of 116.54 Gb of clean data was obtained, with each sample generating more than 11.25 Gb of clean read sequences in RNA-Seq, which were then used for further analysis. Clean reads were mapped to the K326 genome, with approximately 95.70% to 96.09% of clean reads successfully mapped to the genome (Table 3). A total of 1697 differentially expressed genes were identified between WT vs G27, and 2388 differentially expressed genes were identified between WT vs M21 (Fig. 3A). This indicates that there are relatively few differentially expressed genes between G27 and WT, but relatively many differentially expressed genes between M21 and WT. Among these, 1035 genes were upregulated and 662 genes were downregulated between WT vs G27; 1313 genes were upregulated and 1075 genes were downregulated between WT vs M21. There were 301 up-regulated genes and 449 down-regulated genes in G27 vs M21(Fig. 3B). These results indicate that there are significant differences in gene expression between mutant plants and wild-type plants during leaf development.

**Table 3:** Statistical tables of sequencing data and alignment efficiency.

Sample	Clean reads	Q20(20%)	Q30(%)	GC(%)	Contrast ratio(%)
G27_3	85450348	97.61	93.55	43.87	81870733(95.81%)
G27_2	81446076	97.75	93.77	44.00	78036769(95.81%)
G27_1	88298714	97.56	93.43	43.72	84499711(95.7%)
M21_3	87907540	97.84	93.97	43.39	84292419(95.89%)
M21_2	76256784	97.88	93.98	43.84	73022356(95.76%)
M21_1	83200302	97.77	93.83	43.81	79797041(95.91%)
WT_3	84103392	97.66	93.70	44.18	80779276(96.05%)
WT_2	113185342	97.80	93.91	43.94	108690411(96.03%)
WT_1	93567268	97.76	93.85	43.82	89904925(96.09%)

We further screened two groups of differentially expressed genes with opposite expression patterns between G27 and M21: (1) genes significantly upregulated in overexpression mutant plants and downregulated in silenced mutant plants; (2) genes significantly downregulated in overexpression mutant plants and upregulated in silenced mutant plants.Only one genes were identified for the first gene set, while no genes met the screening thresholds for the second group. The expression patterns and functional annotations of this one candidate genes are listed in Supplementary Table 5.

### 2.7 Enrichment analysis of differences gene

GO enrichment analysis was performed on the differentially expressed genes between WT vs G27, and WT vs M21. Differentially expressed genes (DEGs) were screened with the threshold of P-value ≤ 0.05, and the DEGs were defined as the common genes obtained from the intersection of three biological replicates. We found that most of the differentially expressed genes were involved in biological processes. In the enrichment analysis of G27(Supplementary Table 1), the biological process with the highest number of genes was polysaccharide binding and terpenoid metabolic process, with 22 genes, while the biological process with the lowest number of genes was 5-epi-aristolochene synthase activity, with only 8 genes. The biological process with the highest enrichment level was glucan endo-1,3-beta-d-glucosidase activity. In the enrichment analysis of M21(Supplementary Table 2), the isoprenoid biosynthetic process had the highest number of genes, with 34 genes, while the phloem development had the lowest number of genes, with only 6 genes, but the development of the phloem had the highest degree of enrichment. According to the GO enrichment analysis of G27 and M21, the vast majority of genes involved in terpenoid synthesis and glycosidase activity were significantly enriched. Genes enriched in terpenoid synthesis and metabolic pathways displayed the most significant transcriptional variation between G27 and M21, which hints at a potential correlation between terpenoid metabolism and leaf development.

**Fig. 4:**
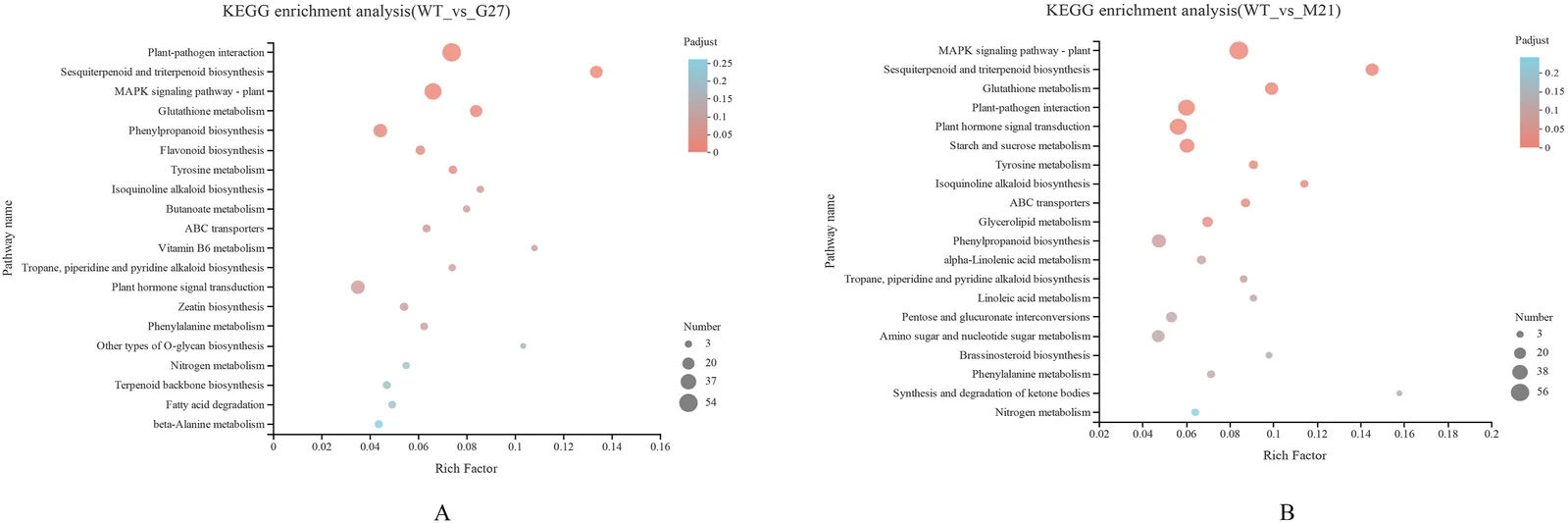
KEGG enrichment analysis of differentially expressed genes between WT vs G27 and WT vs M21. (A) KEGG analysis of differentially expressed genes between WT and G27. (B) KEGG analysis of differentially expressed genes between WT and M21.

Metabolic pathway analysis was performed based on KEGG annotation. The G27 upregulated genes were annotated to 106 pathways, with the highest number of genes enriched in the plant-pathogen interaction pathway, while the sesquiterpene and triterpene biosynthesis pathways showed the highest enrichment levels (Fig. 4A). The M21 upregulated genes were annotated to 113 pathways. The MAPK signaling pathway - plant had the highest number of enriched genes, while the synthesis and degradation of ketone bodies had the highest enrichment level (Fig. 4B). In addition, the most KEGG annotation entries were related to metabolism. Among these differentially expressed genes, 15 were related to carbohydrate metabolism and 5 were related to cell size development. Based on the KEGG analysis, we further studied specific functional genes related to leaf development.

### 2.8 Differential expression of genes related to carbohydrate metabolism in mutant plants

**Fig. 5:**
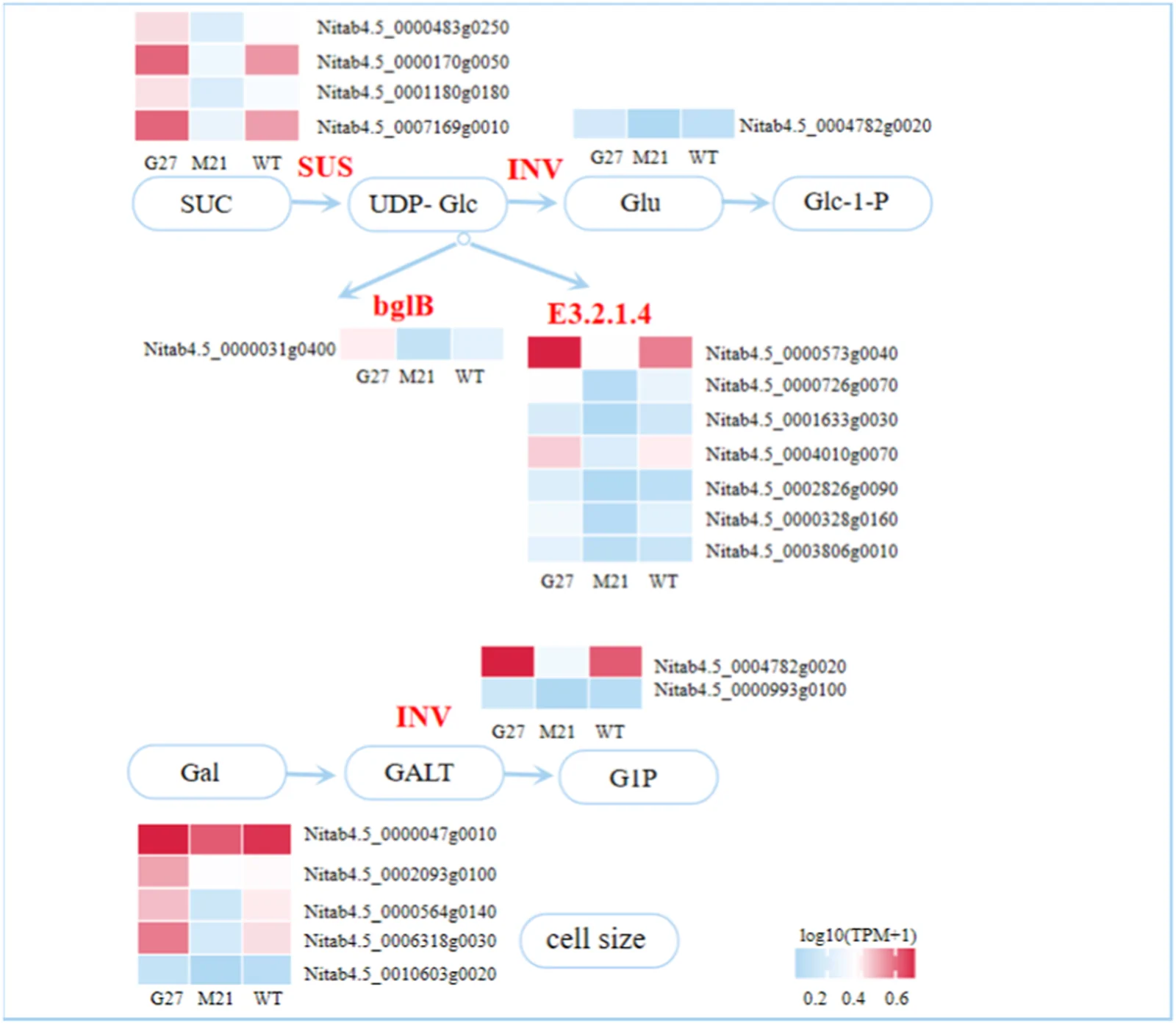
Genes related to starch and sucrose metabolism and galactose metabolism are differentially expressed in wild-type and mutant plants. Note:Cell size:This figure shows the expression fold changes of differentially expressed transcripts annotated as homologs of *RAPTOR, ABP1, ABC1,ABC1* and *T92* genes based on transcriptome sequence analysis.

Combined with functional enrichment analysis, we characterized differentially expressed genes associated with starch, sucrose and galactose metabolism to explore how *NtAN3* affects carbohydrate physiological responses in tobacco leaves(Fig. 5). In the starch and sucrose metabolic pathways, a total of 13 genes were detected, including 4 genes annotated as Sucrose Synthase (SUS), 7 genes annotated as E3.1.2.4, 1 gene annotated as invertase (INV), and 1 gene annotated as phospho-β-glucosidase(bglB).

### 2.9 Overexpression of genes related to cell development during leaf development

In mutant plants, there is a significant enrichment of the GO category related to cell size. In cell size, Nitab4.5_0000564g0140, Nitab4.5_0006318g0030, Nitab4.5_0000047g0010, Nitab4.5_0010603g0020, and Nitab4.5_0002093g0100 showed high expression in G27 (Fig. 5), I've added the expression levels of these genes in G27,M21 and WT to Supplementary Table 7. As summarized in Supplementary Table 7, transcript abundance (average replicate TPM) of the five key candidate genes was substantially higher G27, whereas their expression was distinctly decreased in M21 when compared with wild-type tobacco, indicating that cell size is a key process influencing leaf area. The transcript Nitab4.5_0000047g0010 was annotated as *RAPTOR*, Nitab4.5_0002093g0100 as *ABP1*, Nitab4.5_0000564g0140 and Nitab4.5_0006318g0030 as *ABCB1*, and Nitab4.5_0010603g0020 as *T92*. All of these genes exhibited higher expression levels in G27 relative to M21. The annotation information corresponding to these genes has been added to Supplementary Fig. 6. Implying their potential roles in promoting cell growth.

### 2.10 RT-qPCR

**Fig. 6:**
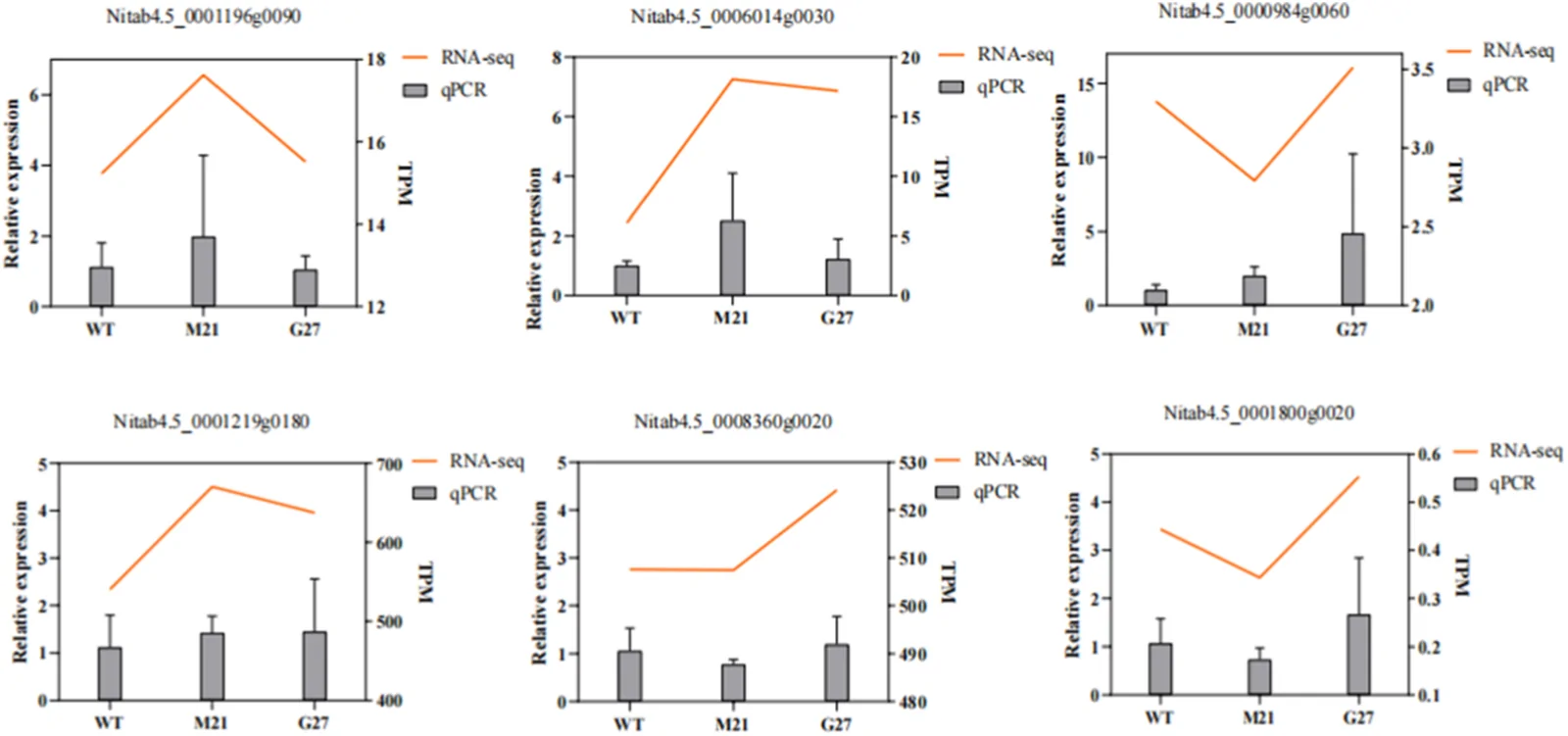
Real-time PCR validation of 6 DEGs.

The expression levels of DEGs were determined by qRT-PCR to verify the reliability of the RNA-seq data (Fig. 6). The results showed that six DEGs exhibited the same trend in both RNA-Seq and qRT-PCR results, indicating that the RNA-seq data were reliable.

## 3 Discussion

Research has demonstrated that the AN3 gene exerts a regulatory influence on leaf growth and development, as evidenced by studies on arabidopsis [5,7] and Chinese cabbage [8]. Transcriptome analysis has effectively revealed the molecular basis of leaf area growth and development in both arabidopsis [23] and Chinese cabbage [8]. However, the molecular basis of leaf area growth and development regulated by the AN3 gene in tobacco has not been reported in the extant literature. The development of upper leaves in tobacco is a critical factor in enhancing both yield and quality [2,3]. From this perspective, comparative transcriptomic analysis between wild-type and mutant plants was performed to characterize downstream transcriptional changes triggered by altered *NtAN3* expression in leaves.

Carbohydrates are important factors in maintaining plant growth and development [24]. Carbohydrate metabolism regulates leaf growth and development by influencing key regulatory genes and enzymes [25]. Through KEGG enrichment analysis of upregulated genes in mutant plants, we found that genes related to carbohydrate synthesis were significantly enriched. To clarify the influences of *NtAN3* on carbohydrate synthesis metabolism in tobacco leaves, we focused on starch and sucrose metabolism, as well as galactose metabolism.

Sucrose is one of the important forms of carbohydrate storage and transport in plants [26]. After being unloaded into storage tissues via the phloem, sucrose is converted into hexoses, which serve as carbon sources and energy for plant utilization [27]. This process is primarily catalyzed by SuS [11] and INV [28].In this study, 13 related genes were identified, which are involved in sucrose and starch metabolism, including 1 INV, 4 SUS, 7 E3.2.1.4, and 1 bgIB. G27 expression levels were higher than WT, and WT expression levels were higher than M21. INV and SUS promote the synthesis of d-fructose-6-phosphate and d-fructose [29], while E3.2.1.4 and bgIB promote d-glucose synthesis [30]. These monosaccharides provide energy for leaf development. This indicates that the overexpression of *AN3* promotes the expression of enzymes involved in sucrose and starch metabolism, and generates secondary effects in tobacco leaves.

The intermediate products produced by galactose metabolism are key products in the synthesis of hemicellulose and pectin [31]. These components give the cell wall elasticity and mechanical strength, directly affecting the expansion capacity of leaf cells. *INV* genes are primarily involved in hexose accumulation and cell proliferation [32] regulating cell elongation through glucose-mediated auxin signaling [33]. In this study,two INV genes involved in the galactose metabolic pathway exhibited higher transcript abundance in G27 relative to M21, suggesting a potential correlation between *NtAN3* accumulation and the expression of these *INV* genes.

Cell size is particularly important for plant growth and development [34]. Studies have reported that the rapamycin (TOR) kinase is a major glucose signaling mediator; it controls plant growth and development by integrating nutritional and energy signals, as well as growth factors, hormones, and environmental signals [35]. The kinase encoded by the *TOR* gene causes cells to enlarge, resulting in larger leaves [36]. Additionally, overexpression of the Arabidopsis *ABP1* gene leads to cell expansion [37]. It has been reported that ABCB proteins are auxin transporters, and CsABCB19 regulates leaf structure by mediating auxin accumulation and transport [38,39]. Research indicates that cotyledon cell expansion depends on the ABCB19 protein to mediate the uptake of auxin in plants [40]. No reports have been made on the association between the T92 gene and leaf development. Transcriptomic analysis revealed altered expression of five genes associated with cell size regulation in mutant plants. However, cytological quantification showed no significant difference in cell size between overexpression and wild-type tobacco. We speculate that transcriptional changes of these genes cannot independently drive cell expansion due to functional redundancy or post-transcriptional regulatory compensation. The enlarged leaf area of *NtAN3* overexpression lines is primarily determined by enhanced cell proliferation rather than cell expansion.

Notably, only one oppositely expressed gene was screened, which indicates that *NtAN3* modulates leaf development via a small set of core downstream targets. Database annotation revealed that this candidate gene is involved in auxin-associated plant growth. We will perform further functional verification on this gene in subsequent experiments to elucidate the regulatory pathway of *NtAN3*.

Previous studies have confirmed that *AN3* functions as a transcriptional co-activator without intrinsic MYB DNA-binding capacity. *AN3* interacts with MYB-containing GRF transcription factors to co-regulate cell cycle-related genes, indirectly facilitating leaf cell proliferation through strengthening GRF transcriptional activity in Arabidopsis [23]. ABP1 forms a complex with the TMK kinase, acting as an extracellular auxin receptor to mediate rapid responses [41]. We reasonably speculate that *AN3* overexpression may elevate *ABP1* expression, further improve auxin response and consequently increase leaf area. According to transcriptome analysis, the gene annotated as an *ABP1* homolog exhibited elevated expression in AN3-overexpressed plants (Fig. 5). We propose that the elevated *ABP1* level enhances auxin response, which consequently increases leaf area. Results showed that the AN3 gene influences the expression of sugar metabolism enzymes(*SUS,* *INV,* *blgB*), whose increased activity raises intracellular glucose concentration, activating the TOR signaling pathway. Studies indicate that the P-glycoprotein encoded by *ABCB1* is involved in the transmembrane transport of auxin [42]. Therefore, we tentatively propose that *AN3* is involved in the regulation of tobacco leaf area through a putative multi-level network consisting of transcription factors, transport proteins and metabolic enzymes.

In this study, there are still several limitations that need to be acknowledged. Firstly, these mutant materials were preliminarily screened and confirmed by phenotypic traits and basic molecular identification only. Further quantitative verification at the transcriptional level is still required. In future research, we will supplement relevant gene expression detection and collect more homologous gene sequences to optimize relevant experimental analysis. Secondly, transcriptome analysis mainly reflects dynamic changes in gene expression and downstream molecular responses during leaf development. The significant enrichment of carbohydrate metabolism pathways indicates that these metabolic processes are closely correlated with leaf growth. At this stage, we cannot fully confirm whether the alteration of carbohydrate metabolism serves as an upstream causal factor initiating leaf area expansion, or is a secondary metabolic response resulting from changes in leaf developmental status. Further genetic and physiological experiments are required to clarify their definite causal relationship in future research. It should be noted that carbohydrate metabolism, terpenoid biosynthesis and MAPK signaling pathways are widely involved in multiple plant growth and metabolic processes, and are not uniquely specific to leaf area development. In this study, we only performed general pathway enrichment analysis, and did not further screen and prioritize core hub genes within these pathways. Further screening of key regulatory factors is required to clarify their specific functions in leaf development.

## 4 Conclusions

This study clarified the role of the *AN3* gene in tobacco by measuring the leaf area of mutant plants. Transcriptome analysis of mutant plants and wild-type plants showed that the expression level of the *AN3* gene affects overall gene expression. There were 2388 differentially expressed genes between M21 and WT, and 1697 differentially expressed genes between G27 and WT. KEGG enrichment analysis of these differentially expressed genes revealed that carbohydrate metabolism pathways and cell size are associated with leaf development. We focused on genes related to sucrose and starch metabolism, galactose metabolism, and cell size, and found that the overexpression of these genes may be associated with increased leaf area. We hypothesize that *NtAN3* may positively modulate the expression of *ABCB*, *ABP1*, *INV*, *SUS* and *bgIB* potentially through the TOR signaling pathway, forming a tentative regulatory cascade linking signal perception to metabolic reprogramming to accelerate leaf cell proliferation and expansion. However, since we have not directly measured TOR activity, endogenous auxin concentrations or downstream signaling outputs in this work, this proposed regulatory network remains a hypothetical framework that requires comprehensive experimental verification in follow-up research. In summary, this study provides new insights into the mechanism by which the *AN3* gene increases tobacco leaf area. However, further studies are needed to elucidate the developmental mechanism of tobacco leaf area by investigating the synergistic effects of the *AN3* gene with these genes.

## 5 Methods

### 5.1 Obtaining plant material

In this research, the tobacco strain NC82 was utilized, sourced from the Guizhou Provincial Key Laboratory for Tobacco Quality Improvement and Efficiency Enhancement at Guizhou University. This tobacco leaf is of the small-leaf type, with narrow and small upper leaves, offering excellent quality and high stability. Mutant samples were generated at the Guizhou Provincial Key Laboratory for Tobacco Quality Improvement and Efficiency Enhancement, while greenhouse field tests were undertaken at the Yangwu Tobacco Research Base affiliated with Guizhou University. The genetic transformation was introduced into the leaves of the NC82 tobacco strain using an Agrobacterium-mediated approach. To generate silenced mutants, an RNAi vector targeting *NtAN3* was constructed. A specific fragment of the *NtAN3* CDS was amplified by PCR and inserted into the pFGC5941 vector in sense and antisense orientations. The recombinant construct was transformed into Agrobacterium tumefaciens GV3101. Genetic transformation of tobacco cultivar NC82 was conducted via the Agrobacterium-mediated leaf disc method. Regenerated resistant seedlings were screened on selective medium, and RT-qPCR was used to identify transgenic lines with significantly suppressed *NtAN3* expression.We ensured that the collection of plant material andexperimental research and field studies on plants compliedwith relevant institutional, national, and internationalguidelines and legislation.

### 5.2 Field experimental design

The field experiment employed a randomized complete block design with three replications. Each plot consisted of four rows, with 15 plants per row, at a spacing of 110 cm × 55 cm. Border rows were established around the experimental area, and the first and last plant in each row were excluded from sampling. Fertilization, plant density, and other crop management practices followed the high-quality tobacco production protocol of the research station.

### 5.3 Agronomic trait measurement

Leaf length and leaf width at the 16th, 17th, and 18th leaf positions were recorded for both mutant and wild-type plants at each developmental stage. We classified plant developmental stages according to morphological characteristics, and the classification criteria were defined as follows: Rosette stage: Plants reach the rosette standard with 12–13 expanded leaves. The ratio of plant width (horizontal growth) to plant height (vertical growth) is approximately 2:1, and the plant shape resembles a hemisphere. Prosperous growing stage: The period spanning from the rosette stage to flower budding stage. Flower buding stage: The stage when flower buds are fully exposed. Central flowers opening stage: The stage when the first central flower of the plant opens. Meanwhile, leaf samples were collected and preserved under two conditions: frozen at appropriate temperatures (frozen samples), and fixed in FAA (formalin-acetic acid-alcohol) solution (FAA-fixed samples) for further analysis.

### 5.4 Statistical analysis and reproducibility

ANOVA followed by Fisher's Least Significant Difference (LSD) post hoc test for multiple comparisons. A P-value < 0.05 was considered statistically significant. The exact P-values are indicated in the figures or figure legends where applicable.

### 5.5 RNA extraction and transcriptome sequencing

At the flower budding stage, three representative plants per line were selected. Leaves from the same position were collected, frozen at −80 °C, and then pooled per line before being sent to Shanghai Majorbio Bio-Pharm Technology Co., Ltd. for total RNA extraction (three replicates per treatment), cDNA library construction, and sequencing. Subsequent DEG screening was accomplished via Majorbio online platform with cutoff criteria: |log_2_(fold change)| ≥ 1 and q-value < 0.05.

### 5.6 Total RNA extraction and cDNA first strand synthesis

Total RNA from the leaves was isolated employing the RNAiso Plus TriZol technique. Subsequently, cDNA was generated following the instructions of the FastKing gDNA Dispelling RT SuperMix (TIANGEN) Reverse Transcription Kit.

### 5.7 Cloning and bioinformatics analysis of the NtAN3 gene

Primers tgtF and tgtR were designed using Primer6.0 software and synthesized by Qingke Biotechnology (Chongqing) Co., Ltd. The PCR output was inserted into the pBWA(V)HS-AN3 vector. Positive colonies were pinpointed using PCR, and the rDNAG1 and rDNAtlt3 products underwent enzymatic digestion followed by sequencing. A similarity comparison between NtAN3 and related genes was undertaken through NCBI. The physicochemical attributes, including hydrophilicity and hydrophobicity, of *Arabidopsis thaliana* AN3 and the acquired glycosidic acid sequences from tobacco homologs were scrutinized with the ProtParam tool.

### 5.8 Genetic transformation and characterization of *NtAN3*

The genetic transformation was executed using Agrobacterium infiltration. From this process, 30 overexpression plants and 6 silenced plants were identified. Following PCR amplification, 24 positive mutant plants were distinguished, comprising 20 overexpression mutants and 4 silenced mutants. For each selected transgenic line, three individual plantlets were taken as biological replicates to perform subsequent physiological and molecular measurements.

### 5.9 Sequencing verification of mutant lines

Genomic DNA was extracted from leaf tissues of mutant plants for PCR amplification. Gene-specific primers were designed to amplify the target fragment of the NtAN3 gene.AN3-F: GAGCCAGAACTCAGGGAAAC.AN3-R:ATCAGCAATAGCAGCAAGGT.

### 5.10 Histological observation and paraffin section analysis

To investigate the cytological differences in leaf structure among wild-type, G27 and M21, mature and fully expanded functional leaves at the same developmental stage(flower building stage) were selected for paraffin section preparation. Leaf tissues were cleaned and dried, and rectangular segments (2 mm × 5 mm) were cut from the middle area of the leaf, avoiding the main vein, to ensure consistent sampling positions.

The collected samples were immediately fixed in FAA solution (50% ethanol, glacial acetic acid, and formaldehyde at a volume ratio of 18:1:1) with a sample-to-fixative ratio of 1:20. Vacuum pumping was applied to remove internal air bubbles and promote full infiltration. Samples were fixed for more than 48 h. After fixation, tissues were rinsed three times with 70% ethanol for 2 h each and immersed in 70% ethanol overnight.

For dehydration, samples were treated with a gradient ethanol series: 70%, 80%, 95%, and two changes of absolute ethanol, with 2 h incubation for each step. Subsequently, tissues were cleared through a gradual xylene-ethanol gradient (1/3 xylene + 2/3 ethanol, 1/2 xylene + 1/2 ethanol, 2/3 xylene + 1/3 ethanol) and two pure xylene treatments. Safranin staining was performed during the clearing process to facilitate tissue localization.

For paraffin infiltration, tissues were incubated in a 1:1 mixture of xylene and paraffin, followed by two rounds of pure paraffin infiltration at 55–60 °C for approximately 3 h each. Paraffin with melting points of 52–54 °C or 56–58 °C was selected according to ambient temperature conditions.

Samples were embedded, trimmed into regular trapezoid blocks, and fixed on paraffin holders. Serial sections were first trimmed at 15–20 μm, and formal histological sections were prepared at a thickness of 8–12 μm. Sections were mounted on glass slides using Haupt's adhesive solution, unfolded in a 36 °C water bath, and dried at 36 °C for more than 24 h.

For staining, slides were deparaffinized and rehydrated through a xylene and ethanol gradient, stained with safranin and fast green, and then dehydrated and cleared gradually. Finally, specimens were mounted with neutral balsam and dried at 36 °C to prepare permanent sections.

All sections were observed and photographed using an Olympus light microscope. Leaf thickness measurements were performed on paraffin cross-section images captured under a 20× microscope objective. For mesophyll cell number counting and single cell size quantification, micrographs were acquired at a uniform 40× objective magnification. A fixed frame with identical pixel dimensions was used to delineate the statistical region on each 40× image, and all cells inside the equal-area frame were counted manually; meanwhile, the size of individual cells within the framed area was measured. Uniform magnification and standardized statistical frames ensured comparable data across wild-type and mutant plants.

### 5.11 Real-time fluorescence quantitative analysis of the NtAN3 gene

Total RNA was isolated from tobacco leaves of overexpressed, silenced, and wild type. The concentration and purity of the retrieved RNA were assessed using Nanodrop2000 and further validated through gel electrophoresis. A 1291-bp fragment of NtAN3 was amplified for gene cloning, and a separate short amplicon suitable for real-time PCR (100–150 bp) was used for expression quantification. The sequences of the synthesized primers are shown in Supplementary Table 6.Fluorescence experiments were conducted in line with the Talent qPCR PreMix (SYBR Green) kit's guidelines. The *NtAN3* relative expression level was gauged using a fluorescence quantitative PCR machine. Gene expression was quantified employing the 2–ΔΔCt technique [43]. The Actin gene functioned as the reference gene, and each gene's evaluation was repeated thrice. qRT-PCR specific primers targeting the candidate genes were formulated using Primer6.0 and Primer-BLAST (https://www.ncbi.nlm.nih.gov/tools/primer-blast/) available on NCBI. DynaPro Bio (Chongqing) Co. The fluorescence quantitative PCR system included 2×Talentq PCR PreMix 10 μL, 10 μM PrimerF 0.6 μL, 10 μM PrimerF 0.6 μL, cDNA 2 μL, and RNase-Free ddH2O to make final volume 20 μL. The PCR reaction settings were: an initial phase at 94 °C for 3 min, followed by 40 cycles each at 95 °C for 5 s, 50–60 °C for 10 s, and 72 °C for 15 s.

## Funding

This work was supported by Guizhou Provincial Science and Technology Department Project (Grant No. Qiankehe Platform ZSYS[2025]028); Guizhou Tobacco Company Project (Grant No. 2025XM05); and Guizhou Postgraduate Innovation Fund Project (Grant No. Qianjiaohe YJSKYJJ[2021]070).

## Declaration of competing interests

The author declares no conflicts of interest that could potentially affect the research results.

## Data Availability

Data will be made available on request.
