# Supplementary material for: The NtAN3 gene plays a key role in increasing tobacco leaf area
Source: Biochem Biophys Rep. 2026 Aug 13;47:102749. doi: 10.1016/j.bbrep.2026.102749 (PMC13499148; doi:10.1016/j.bbrep.2026.102749)
Supplement: Supplementary file 1 [file mmc2.docx]

**Supplementary Fig 1**

Upper panel: sequencing result from the NtAN3-overexpressing transgenic line; Lower panel: sequencing result from the NtAN3-silenced transgenic line. The chromatograms verify the sequence of the amplified fragment of native NtAN3 coding sequence.

**Supplementary Fig 2**

**M21	WT	G27**

Determination results of target gene expression in mutant plants

Note: different lowercase letters represent P < 0.05, with significant difference

**Supplementary Fig 3**

Domain analysis

Note: Purple bars indicate conserved amino acid segments; grey lines represent non-conserved regions of the polypeptide chain.

**Supplementary Fig 4**

Tertiary structure analysis

Note: The three-dimensional structural models of AtAN3 and NtAN3 proteins were predicted using the Phyre2 online server (http://www.sbg.bio.ic.ac.uk/phyre2/html/page.cgi?id=index).

**Supplementary Fig 5**

A. **Blade thickness:M21**

A. **Blade thickness:G27**

A. **Blade thickness:WT**

**B.Cell size:M21**

**B.Cell size:G27**

**B.Cell size:WT**

**C.Cell number:M21**

**C.Cell number:G27**

**C. Cell number:WT**

**Supplementary Fig 6**

**G27	M21	WT**
